# Morphological Abnormalities in Early-Onset Schizophrenia Revealed by Structural Magnetic Resonance Imaging

**DOI:** 10.3390/biology12030353

**Published:** 2023-02-23

**Authors:** Jacob Levman, Priya Kabaria, Masahito Nangaku, Emi Takahashi

**Affiliations:** 1 Department of Computer Science, St. Francis Xavier University, Antigonish, NS B2G 2W5, Canada; 2 Athinoula A. Martinos Center for Biomedical Imaging, Massachusetts General Hospital, Harvard Medical School, Boston, MA 02129, USA; 3 Nova Scotia Health Authority, Halifax, NS B3H 1V7, Canada; 4 Department of Medicine, Boston Children’s Hospital, Harvard Medical School, Boston, MA 02215, USA

**Keywords:** magnetic resonance imaging, schizophrenia, cortex

## Abstract

**Simple Summary:**

There is much we do not know about how the brain presents in early-onset schizophrenia. This study involves investigating the brains of patients with early-onset schizophrenia using magnetic resonance imaging. Results demonstrate abnormal curvature of the surfaces of various regions of the brain. These results imply that abnormal neurodevelopment associated with early-onset schizophrenia can be characterized with structural magnetic resonance imaging.

**Abstract:**

Schizophrenia is a pathological condition characterized by delusions, hallucinations, and a lack of motivation. In this study, we performed a morphological analysis of regional biomarkers in early-onset schizophrenia, including cortical thicknesses, surface areas, surface curvature, and volumes extracted from T1-weighted structural magnetic resonance imaging (MRI) and compared these findings with a large cohort of neurotypical controls. Results demonstrate statistically significant abnormal presentation of the curvature of select brain regions in early-onset schizophrenia with large effect sizes, inclusive of the pars orbitalis, pars triangularis, posterior cingulate cortex, frontal pole, orbital gyrus, lateral orbitofrontal gyrus, inferior occipital gyrus, as well as in medial occipito-temporal, lingual, and insular sulci. We also observed reduced regional volumes, surface areas, and variability of cortical thicknesses in early-onset schizophrenia relative to neurotypical controls in the lingual, transverse temporal, cuneus, and parahippocampal cortices that did not reach our stringent standard for statistical significance and should be confirmed in future studies with higher statistical power. These results imply that abnormal neurodevelopment associated with early-onset schizophrenia can be characterized with structural MRI and may reflect abnormal and possibly accelerated pruning of the cortex in schizophrenia.

## 1. Introduction

Schizophrenia is a neurological disorder with those affected experiencing hallucinations, thought disorders, delusions, and motivational problems. Early-onset schizophrenia (EOS) is characterized by the onset of symptoms at age 19 or earlier, with a subgroup of this population, with symptom onset at age 12 or earlier, referred to as either very early-onset schizophrenia, or childhood-onset schizophrenia. Although early twentieth-century post-mortem analyses did not discover anatomical features specific to schizophrenia [1,2,3,4], the first computer-assisted tomography (CT) analysis observed enlarged lateral ventricles [5]. Magnetic resonance imaging (MRI) and CT-based studies have more recently confirmed the previously observed ventricular enlargement, and ventricular enlargement’s presence in first-episode medication-naïve patients is an accepted feature of schizophrenia [6]. MRI technology has successfully been used to regionally evaluate abnormal development across the brain, including investigations of the prefrontal, orbitofrontal, medial temporal, and parietal lobes, as well as the ventricles [7,8,9].

The analysis of individuals with schizophrenia by MRI has been investigated across many studies; however, research has been limited with respect to the age ranges of the participants, as well as in terms of the distributed regions of the brain analyzed [10,11,12]. MRI studies focused on early-onset schizophrenia have reported abnormalities of the cerebral gray matter [13], specifically regarding robust cortical gray matter loss during adolescence [14,15]. Specific abnormalities have been observed in the anterior cingulate [16], hippocampus [17,18], temporal lobe [19], cerebellum [20], superior temporal gyrus [21], corpus callosum [22], and the ventricles [23]. Analyses have indicated that “it is probable that neuroanatomical cerebral abnormalities present prior to disease onset play an etiopathogenic role in the development of schizophrenia” [24], which implies that measurements extracted from brain MRI examinations may support the eventual non-subjective characterization of schizophrenia, as opposed to the symptom-based diagnoses currently performed as outlined in the Diagnostic and Statistical Manual of Mental Disorders (DSM). Schizophrenia is a very rare condition; as such, thorough assessment of extractable measurements across patients and age groups, towards accurate characterization of the presentation of the brain, is a major research challenge.

Although a wide variety of methodologies have been used for the analysis of brain development in schizophrenia from MRI examinations [17,18,19,20,21,22,23], MRI-based schizophrenia studies have been enhanced by technological innovations such as FreeSurfer, which automatically extracts biomarkers of potential interest [25]. Neurological image analysis technologies, such as FreeSurfer, can aid in the extraction of measurements across a patient’s brain. The technology performs volume-, as well as surface-based analyses, and models pial and gray/white matter surfaces, providing a variety of biomarker measurements, such as surface areas, cortical thicknesses, and regional volumetrics. Studies of schizophrenia combining FreeSurfer and MRI technology have reported interdependence between altered gray matter structure and abnormal white matter connections [26], abnormal cortical folding [27], subcortical volume abnormalities [28,29,30,31,32,33], gyrification abnormalities [34,35], reduced cortical thickness [36,37,38], reduced hippocampal volume [39], reduced dorso-lateral prefrontal cortex [40], reduced cerebellar cortical volume [41], and subcortical brain volume abnormalities [42] potentially associated with schizophrenia. These findings from several studies imply that a heterogeneous collection of abnormalities can be detected in schizophrenic populations, and that biomarker extraction technology may be able to contribute to the characterization of regional neurological abnormalities.

In this study, we hypothesized that a thorough investigation (inclusive of all brain regions analyzed by FreeSurfer) would reveal developmental abnormalities characterized by cortical thicknesses (including within-region cortical thickness variability), surface areas, surface curvatures, and regional volumetrics in early-onset schizophrenia. We further hypothesized that inclusion of a thorough set of biomarkers with strict controls for the multiple comparisons problem would result in the observation of novel findings that may help characterize the abnormal development associated with early-onset schizophrenia. We are hopeful that, eventually, measurements extracted from brain MRI examinations may provide more specific characterization of schizophrenia in a non-subjective manner than is currently possible with symptom-based diagnoses.

## 2. Materials and Methods

### 2.1. Participants, Data Acquisition, and Preprocessing

Following approval by BCH’s Institutional Review Board (IRB-P00032682—informed consent was waived due to the lack of risk to participants included in this retrospective analysis), a retrospective review of clinical imaging was performed and patient examinations were accessed through the Children’s Research and Integration System [43]. Imaging was performed on a suite of 3T Siemens Skyra scanners at BCH. All examinations including non-contrast-enhanced volumetric T1 acquisitions were included for further analysis. This was a retrospective review of clinical data; as such, there was variability in the pulse sequences used to acquire these T1 volumetric exams. Strengths and weaknesses of this approach are addressed in the Discussion. The spatial resolution was, on average, 1 mm in each direction. Each T1 structural examination was processed with FreeSurfer technology [25], using recon-all, which aligns each input examination to all supported atlases. Each FreeSurfer output T1 structural examination was visually displayed with label map overlays and manually inspected for regional segmentation quality. FreeSurfer results that were observed to fail were excluded from this analysis (i.e., FreeSurfer regions of interest (ROIs) that do not align to the MRI and exams in which major problems were observed with an ROI, for example, a cerebellar segmentation extending far beyond the extent of the cerebellum). This resulted in a collection of 993 MRI examinations that passed quality control in our neurotypical cohort [44], and 23 MRI examinations that passed quality control in our early-onset schizophrenia cohort. Our early-onset schizophrenia cohort had an age range from 7.4 to 23.2 years (mean: 14.1 years old). Our neurotypical cohort consisted of a broader range of ages, but ages beyond the age range represented in our early-onset schizophrenia group were excluded from the group-wise analyses presented herein, resulting in 734 included examinations (mean: 13.5 years old). Demographic information of our study participants is presented in Table 1.

### 2.2. Statistical Analysis

This study included the acquisition of 662 regionally distributed cortical thickness measurements, 448 regionally distributed surface area measurements, 1320 surface curvature measurements, and 463 regionally distributed volume measurements per imaging examination (across both left and right hemispheres), as extracted by FreeSurfer, using recon-all to analyze each MRI exam with all available atlases. This includes the Desikan-Killiany atlas, from which sets of statistical biomarkers labeled aparc and w-g.pct are computed; the Desikan–Killiany–Tourville atlas, from which a set of statistical biomarkers labeled aparc.DKTatlas40 are computed; the Destrieux atlas, from which a set of statistical biomarkers labeled aparc.a2009s are computed; the subcortical atlas, from which a set of statistical biomarkers labeled aseg are computed; the Brodmann’s Areas atlas, from which sets of statistical biomarkers labeled BA and BA.thresh are computed; and finally, the set of statistical biomarkers labeled wmparc are derived from a white matter parcellation established by labeling the white matter based on the Desikan–Killiany label of the closest point in the cortex. Each of the following sets of statistical biomarkers (aparc, aparc.DKTatlas40, aparc.a2009s, BA, BA.thresh, entorhinal_exvivo), include a single measurement of each of surface area, gray matter volume, average cortical thickness, standard deviation of cortical thickness, mean curvature, Gaussian curvature, folding index, and curvature indices for each supported region of interest. The w-g.pct set of biomarkers includes surface area measurements for each supported region of interest. The aseg and wmparc sets of biomarkers include volume measurements for each supported region of interest. The cortical thickness biomarkers represent the extraction of measurements of both the average and standard deviation of within-region cortical thicknesses across grey matter regions (in mm). The variability (as measured with the standard deviation) of within-region cortical thicknesses provides a single measurement of cortical thickness variability localized to a single brain region within a single study participant. Cortical surface area is included (in mm^2^), as are regional volumetrics (in mm^3^). Surface curvature measurements include the folding index, a single number summarizing the overall amount of folding on a cortical surface (a unitless measure); the intrinsic curvature index, a “natural” index (also a unitless measure); the mean curvature, which is the average of the two principal curvatures (in mm^−1^); and the Gaussian curvature, which is the product of the two principal curvatures (in mm^−2^). Higher curvature values imply that brain folding is “sharper” in at least one direction. Effect sizes were evaluated with Cohen’s d statistic, in which positive/negative values indicate higher/lower average values in the schizophrenia population relative to the neurotypical population. A *p*-value based on the standard *t*-test [45] for two groups of samples was computed to assist in the assessment of group-wise differences between the two populations under consideration. The *p*-value is an established method to demonstrate that it is unlikely for study findings to be the result of random chance, and Cohen’s d statistic is the most common method to assess effect sizes. This yielded a total of m = 662 + 448 + 1320 + 463 = 2893 measurements included for group-wise comparisons. The Bonferroni correction for the multiple comparisons problem was employed, resulting in a threshold for statistical significance of *p* < 0.05/m = 0.05/2893 = 1.73 × 10^−5^.

A multivariate regression statistical model was constructed (using MATLAB’s mvregress function), adjusting each measurement in order to control for group-wise differences in gender, age, and the estimated total intracranial volume. This model was used to adjust each biomarker measurement, in order to evaluate whether group-wise differences between our pathological and neurotypical populations are the result of age, gender, or brain volume effects.

## 3. Results

**Figure 1:**
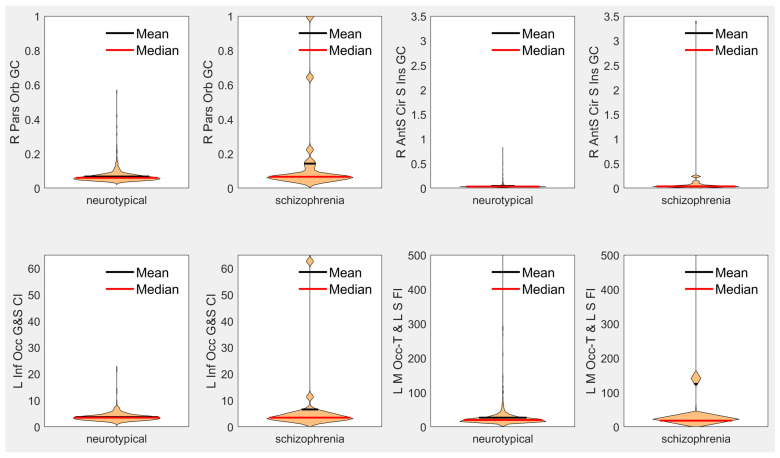
**Violin plots of leading measurements from Table 2 of each type.** **R = right, L = left, Orb = Orbitalis, GC = Gaussian Curvature, AntS = Anterior Segment, Cir = Circular, S = Sulcus/Sulci, Inf = Inferior, Occ = Occipital, G = Gyrus, CI = Curvature Index, FI = Folding Index, M = Medial, Occ-T = Occipito-Temporal, L = Lingual.**

Results demonstrate statistically significant abnormal presentation of the curvature of select regions in early-onset schizophrenia with large effect sizes, inclusive of the right pars orbitalis, the left subcentral gyrus, right pars triangularis, right posterior cingulate, right orbital gyrus, right frontal pole, left inferior occipital gyrus, right lateral orbitofrontal gyrus, as well as in the left medial occipito-temporal and lingual sulci and in the right insular sulci (see Table 2 for more detail). Leading findings from Table 2 are visualized with violin plots (https://www.mathworks.com/matlabcentral/fileexchange/45134-violin-plot accessed on 18 January 2023) in Figure 1. No statistically significant differences were observed between our neurotypical and schizophrenia populations in terms of estimated total intracranial volume, total gray matter volume, mean thickness across the whole left hemisphere, nor mean thickness across the whole right hemisphere.

Our cohort of patients with early-onset schizophrenia was small (just 23 examinations), and we tested for many potential regional T1-derived biomarkers (*n* = 2893). In order to help ensure that we do not report statistically significant findings that would not hold in future studies, we employed the Bonferroni correction, the strictest method for the multiple comparisons problem. This may result in our analysis including biomarkers of interest that do not reach statistical significance while potentially still being of interest, provided that the effect sizes hold in larger studies with improved statistical power. These findings include abnormal reductions of cortical thickness variability in the right lingual gyrus (d = −0.76, *p* < 0.0003), the horizontal ramus of the anterior segment of the right lateral sulcus (d = −0.71, *p* < 0.0008), the left middle frontal sulcus (d = −0.70, *p* < 0.0009), the left cuneus (d = −0.68, *p* < 0.002), left Brodmann’s visual area V2 (d = −0.67, *p* < 0.002), the right postcentral gyrus (d = −0.62, *p* < 0.004), right parahippocampal gyrus (d = −0.61, *p* < 0.004), and the right pars triangularis (d = −0.61, *p* < 0.004). We also observed reduced average cortical thickness values that did not reach our stringent standard for statistical significance in the right medial occipito-temporal and lingual sulci (d = −0.70, *p* < 0.001), the right parahippocampal gyrus (d = −0.66, *p* < 0.002), the lateral aspect of the right superior temporal gyrus (d = −0.64, *p* < 0.003), left Brodmann’s middle temporal area (d = −0.61, *p* < 0.004), and the right lateral occipital gyrus (d = −0.61, *p* < 0.004).

We observed reductions in regional surface areas that did not reach our stringent standards for statistical significance in the left lingual gyrus (d = −0.64, *p* < 0.003), right transverse temporal gyrus (d = −0.63, *p* < 0.004), and the right anterior transverse temporal gyrus (of Heschl specifically) (d = −0.60, *p* < 0.005). We observed reductions in regional volumetric measurements that did not reach our stringent standards for statistical significance in the left parahippocampal gyrus (d = −0.75, *p* < 0.0004), right transverse temporal gyrus (d = −0.71, *p* < 0.0008), left lingual gyrus (d = −0.66, *p* < 0.002), the right anterior transverse temporal gyrus (of Heschl specifically) (d = −0.65, *p* < 0.003), and the central segment of the corpus callosum (d = −0.61, *p* < 0.004). Corresponding *p*-values and d statistics were provided for potentially interesting findings that did not reach our stringent standard for statistical significance so that they can be compared with future studies and support further investigation of regional abnormalities in early-onset schizophrenia.

## 4. Discussion

Our primary findings include increased regionally distributed surface curvature measurements in early-onset schizophrenia relative to neurotypical controls. This includes abnormally increased surface curvature in the right pars orbitalis, left subcentral gyrus, right pars triangularis, right posterior cingulate, right orbital gyrus, right frontal pole, left inferior occipital gyrus, right lateral orbitofrontal gyrus, as well as in the left medial occipito-temporal and lingual sulci, and right insular sulci (see Table 2 for more detail). Abnormalities of the pial surface curvature have previously been reported in schizophrenia [46,47,48]; however, no pial surface curvature measurement abnormalities were identified in a young adult population with first-episode schizophrenia [49]. A regionally focused study reported abnormally increased gyrification in the right parahippocampal–lingual cortex area [50], which is supported by our findings of an abnormally increased lingual sulcus folding index in schizophrenia (d = 1.06 see Table 2), as well as our findings of increased cortical thickness variability (which is inevitably linked with the conformation of the cortical surface) in the right parahippocampal (d = −0.61) and right lingual (d = −0.76) cortices, findings that need confirmation in future studies of higher statistical power. 

Changes in cortical surface curvature can be caused by a variety of factors. Identified mechanisms that may contribute to models of cortical folding include the skull constraining brain growth causing compressive buckling, tension in axons pulling cortical regions together to form gyri, greater expansion of outer cortical layers relative to inner layers causing folding by mechanical instability, and programmed patterns of growth causing more neurons to grow into gyri rather than sulci [51]. The brain is a complex structure; as such, we may expect cortical folding to proceed based on multiple underlying contributing factors. It is also possible that irregular and possibly accelerated pruning contributes to observed abnormalities in schizophrenia, as the removal of tissue from the brain through pruning may result in reduced structural support to the conformation of the cortical surface, potentially resulting in increased curvature. Pruning irregularities have previously been hypothesized as potentially characterizing schizophrenia, and it was proposed that the phenomenon affects the prefrontal cortex [52,53,54]. Our findings of increased curvature (with large effect sizes) in the frontal pole, the pars orbitalis, orbital gyrus, and the pars triangularis (see Table 2) are supportive of Feinberg’s theory of irregular pruning of the prefrontal cortex in schizophrenia [52]. Furthermore, our findings potentially imply that irregular pruning in schizophrenia might extend to additional regions of the brain, such as the subcentral gyrus, the occipital gyrus, the lingual gyrus, and the cingulate, which would need to be confirmed in future studies with higher statistical power.

We were also able to confirm known ventricular enlargement effects in the left lateral ventricle (d = 0.54), right lateral ventricle (d = 0.49), third ventricle (d = 0.49), fourth ventricle (d = 0.46), and the fifth ventricle (d = 0.28), in agreement with historical observations of increased ventricle volumes in schizophrenia [5,6]. This represents clinical imaging validation of known ventricular enlargement effects in schizophrenia.

Figure 1 demonstrates that the differences between our early-onset schizophrenia cohort relative to our neurotypical cohort observed in our leading biomarkers are based on a subset of the schizophrenia population exhibiting large differences from our neurotypical baseline. These findings are supportive of the theory that schizophrenia is a fundamentally heterogeneous condition when observed as a whole. Heterogeneity in schizophrenia represents a major analytic challenge due to the fact that the patient populations are difficult to image, in part because of the low prevalence of the condition. Indeed, early research on schizophrenia has suggested that heterogeneity present in schizophrenia may be the result of divisions within the population based on precipitating factors that distinguish between organic and idiopathic manifestations of the condition, with differences between the two supported by evidence of differences in symptoms and from family studies [55]. From early on in the analysis of schizophrenia heterogeneity, the available evidence for differentiating between subtypes of schizophrenia was inconclusive [55]. Subsequent research has focused on the use of cluster analysis to differentiate between potential subtypes of schizophrenia [56]; however, no consensus exists on schizophrenia subtype classification, and this initial effort only identified two potential clusters or subtypes. Subsequent research, performing more sophisticated cluster analyses, summarized in review [57], has indicated the potential for four to five inherent clusters or subtypes [57], which vary in level or pattern of performance. It is interesting to note that although this type of research typically generated potentially meaningful subtypes, there was often “little correspondence between subtyping systems based upon cognitive function and those based upon symptom profile” [57]. These analyses indicate that “there may be different mechanisms for producing cognitive and symptomatic heterogeneity” [57] in schizophrenia. Indeed, much more recent research has investigated brain heterogeneity in schizophrenia and its association with polygenic risk, and “may reflect higher sensitivity to environmental and genetic perturbations in patients” [58], and additional studies have also reported higher brain structural heterogeneity in schizophrenia [59,60]. Our findings are supportive of these theories of structural heterogeneity in early-onset schizophrenia, as illustrated by the wide distribution of surface curvature and folding index biomarkers in our schizophrenia cohort (see Figure 1).

Limitations of this study include the fact that it was performed on a relatively small cohort of early-onset schizophrenia patients, and the mixed pulse sequence protocol that was relied upon in order to investigate a real-world clinical population. Previous analyses have discussed the limitations of mixed pulse sequence study designs [61]. Patient medication status was not available, and so the potential effects of medications could not be assessed as part of this study. Strengths include the large number of samples in our neurotypical cohort, providing a statistically reliable baseline from which to observe deviations in the schizophrenia cohort. By investigating the clinical presentation of early-onset schizophrenia, we were also able to clinically validate findings from previous analyses.

Future work will look at improving our ability to discriminate between schizophrenic and neurotypical participants with the help of additional MRI modalities, such as diffusion tractography and fMRI, as well as with multivariate analysis techniques. It is hoped that these research avenues will assist towards a better understanding of schizophrenia as well as improved characterization, diagnosis, and classification of the disorder into subtypes. 

## 5. Conclusions

We performed a brain MRI-based morphological analysis of patients with early-onset schizophrenia compared with a large population of neurotypical controls. Results demonstrated statistically significant abnormal presentation of the curvature of select brain regions in early-onset schizophrenia with large effect sizes, inclusive of the pars orbitalis, pars triangularis, posterior cingulate cortex, frontal pole, orbital gyrus, lateral orbito-frontal gyrus, inferior occipital gyrus, as well as in medial occipito-temporal, lingual, and insular sulci. We also observed reduced regional volumes, surface areas, and variability of cortical thicknesses in early-onset schizophrenia relative to neurotypical controls in the lingual, transverse temporal, cuneus, and parahippocampal cortices that did not reach our stringent standard for statistical significance and should be confirmed in future studies with higher statistical power. These results imply that abnormal neurodevelopment associated with early-onset schizophrenia can be characterized with structural MRI and may reflect abnormal and possibly accelerated pruning of the cortex in schizophrenia.

## Figures and Tables

**Table 1:** Demographic Information of Study Participants.

Demographic Feature	Schizophrenia	Neurotypical
Minimum age	7.44 years	7.45 years
Maximum age	23.25 years	22.63 years
Average age	14.07 years	13.49 years
Female Count	10	472
Male Count	13	262

**Table 2:** Effect sizes for regions exhibiting statistically significant group-wise differences.

Regional Gaussian Curvature Measurements	M (Std) Sc	M (Std) H	*p*-Value	Cohen’s d
Right Pars Orbitalis Gaussian Curvature (aparc.DKTatlas40)	0.14 (0.22)	0.07 (0.04)	2.82 × 10^−11^	1.39
Right Anterior Segment of the Circular Sulcus of the Insula Gaussian Curvature (aparc.a2009s)	0.21 (0.70)	0.04 (0.06)	1.52 × 10^−8^	1.19
Left Subcentral Gyrus and Sulcus Gaussian Curvature (aparc.a2009s)	1.47 (6.71)	0.08 (0.17)	2.31 × 10^−8^	1.17
Right Posterior-Dorsal Cingulate Gyrus Gaussian Curvature (aparc.a2009s)	1.19 (5.23)	0.11 (0.26)	5.48 × 10^−8^	1.14
Right Posterior Cingulate Gaussian Curvature (aparc.DKTatlas40)	0.60 (2.21)	0.11 (0.25)	3.52 × 10^−7^	1.07
**Regional Curvature/Folding Index Measurements**				
Left Inferior Occipital Gyrus and Sulcus Curvature Index (aparc.a2009s)	6.58 (12.38)	3.71 (1.60)	3.48 × 10^−7^	1.07
Left Medial Occipito-Temporal Sulcus and Lingual Sulcus Folding Index (aparc.a2009s)	125.00 (481.60)	26.76 (39.45)	4.32 × 10^−7^	1.06
Right Frontal Pole Curvature Index (aparc)	4.59 (8.95)	2.46 (1.32)	6.96 × 10^−7^	1.04
Right Orbital Gyrus Curvature Index (aparc.a2009)	33.32 (90.50)	13.29 (11.12)	7.36 × 10^−7^	1.04
Right Lateral Orbitofrontal Curvature Index (aparc.DKTatlas40)	39.46 (91.72)	18.43 (14.80)	4.03 × 10^−6^	0.97
Right Pars Triangularis Curvature Index (aparc.DKTatlas40)	10.96 (21.40)	5.68 (4.07)	5.07 × 10^−6^	0.96
Right Pars Orbitalis Curvature Index (aparc.DKTatlas40)	5.13 (5.74)	3.34 (1.71)	1.51 × 10^−5^	0.91

Positive Cohen’s d values indicate that the schizophrenic population has a higher average measured value. M is mean, Std is standard deviation, *p* is *p*-value, d is Cohen’s d statistic, Sc is schizophrenia, H is healthy.

## Data Availability

The data used in this analysis are private data acquired at Boston Children’s Hospital.
